# Hypoxia-responsive gene *F3* Promotes GBM Cell Proliferation and Migration through Activating NF-κB/p65 Signaling Pathway

**DOI:** 10.7150/jca.97357

**Published:** 2024-06-17

**Authors:** Aixin Yu, Yiqi Wang, Jun Qin, Junrong Lei, Wendai Bao, Zhiqiang Dong

**Affiliations:** 1 College of Biomedicine and Health, College of Life Science and Technology, Huazhong Agricultural University, Wuhan, Hubei 430000, China.; 2 Hubei Clinical Research Center of Central Nervous System Repair and Functional Reconstruction, Taihe Hospital, Hubei University of Medicine, Shiyan, Hubei 430000, China.; 3 Department of Neurosurgery, Taihe Hospital, Hubei University of Medicine, Shiyan, Hubei 430000, China.; 4 Central Laboratory, Hubei Cancer Hospital, Wuhan, Hubei 430000, China.

**Keywords:** GBM, *F3*, proliferation, migration, NF-κB

## Abstract

**Background:** Glioblastoma multiforme (GBM) is the most common malignant form of glioma, but the molecular mechanisms underlying the progression of GBM in hypoxic microenvironment remain elusive. This study aims to explore the pathological functions of hypoxia-responsive genes on GBM progression and its downstream signaling pathways.

**Methods:** RNA-seq was performed in normoxic and hypoxic U87 cells to identify the differentially expressed genes (DEGs) under hypoxia. The mRNA expression levels of hypoxia-responsive gene *F3* in glioma clinical samples were analyzed according to the transcriptional information from CGGA, TCGA and Rembrandt databases. EdU, transwell and wound-healing assays were conducted to evaluate the pathological functions of *F3* on GBM proliferation and migration under hypoxia. RNA-seq and gene set enrichment analysis were conducted to analyze the enriched pathways in LN229 cells overexpressed F3 compared to controls. GBM cells were treated with NF-κB inhibitor PDTC, and cell experiments were performed to evaluate the effects of PDTC on OE-F3-LN229 and OE-F3-U87 cells. Western blot was performed to validate the downstream pathways.

**Results:** *F3* was identified as a hypoxia responsive gene in GBM cells. The mRNA expression level of *F3* was negatively correlated with the overall survival of glioma patients, and significantly increased in grade IV and GBM than lower grade or other histology of glioma. Overexpression of *F3* enhanced the proliferation and migration of hypoxic U87 and LN229 cells, while knockdown inhibited them. In OE-F3-LN229 cells, the NF-κB pathway was activated, with an increased level of phosphorylated p65. PDTC treatment effectively rescued the enhanced proliferation and migration of OE-F3-LN229 cells under hypoxia, indicating that the effect of *F3* on GBM progression is probably dependent on the NF-κB pathway.

**Conclusion:** Hypoxia-induced *F3* activates NF-κB pathway through upregulation of the phosphorylated p65, thus promoting the proliferation and migration of GBM cells under hypoxia, which might be a potential therapeutic target for GBM treatment.

## Introduction

Glioblastoma multiforme (GBM) is the highest grade of gliomas and the most common primary brain tumor in adults. In China, 45.25% of gliomas were diagnosed as GBM from 2011-2017 1. Although great progress has been achieved in the field of GBM treatment such as surgery, chemotherapy, radiotherapy, immunotherapy, malignant proliferation and infiltration to adjacent normal brain tissues mean that GBM is still a high-risk central nervous system tumor with a median survival of < 2 years 2, 3. Hence, exploration of the underlying molecular mechanisms associated with glioma cell progression is in an urgent need.

Hypoxia induced by excessive cell proliferation and poorly organized tumor vasculature is a common microenvironment in solid tumors including GBM 4, 5. Low oxygen concentration in brain results in difficulties for GBM treatment 6. For example, GBM cells with hypoxic microenvironment are usually away from blood vessels, thus leading to worse access of chemotherapy to tumors 7. Hypoxia also promotes the expression of temozolomide (TMZ) resistance gene 8, 9. Hypoxia-induced gene *SHOX2* significantly enhances the methylation of MGMT promoter, which causes TMZ resistance in GBM 10. Moreover, hypoxia prevents the repair of oxidative DNA damage, contributing to radio resistance in cancer treatment 11. Therefore, it is crucial to understand the mechanisms underlying the adaption of GBM cells to hypoxia.

F3 (also known as tissue factor or CD142) was originally identified as a cell surface receptor that initiates blood coagulation 12. Recent studies revealed that F3 could trigger multiple oncogenic pathways in the therapy-induced tumor cell senescence setting in various cancers 13. In colorectal cancer, *F3* is a potential oncogene that is negatively correlated with the vital tumor suppressor p53 14. The concomitant inactivation of E-cadherin as an EMT marker is correlated with highly expressed *F3* in breast cancer 15, 16. Besides, F3 is also reported as a critical regulator of radiation therapy-induced glioblastoma remodeling 17. However, its role in modulating GBM progression under hypoxia remains unknown.

In this study, we identified *F3* as a hypoxia-responsive gene in GBM cells through RNA-seq and revealed that overexpression of *F3* could promote GBM proliferation and migration under hypoxia. Mechanistically, overexpressed *F3* induced up-regulation of phosphorylated p65 and activation of NF-κB pathway. Administration of NF-κB inhibitor could effectively rescue the enhanced proliferation and migration caused by *F3* overexpression. All these findings indicated that *F3* plays an oncogenic role in GBM cells under hypoxia through activation of NF-κB, and may provide a potential therapeutic target for GBM treatment.

## Materials and Methods

### Cell culture

Human GBM cell lines (DBTRG, LN229, U251 and U87) and HEK 293T were purchased from American Type Culture Collection (ATCC). All cells were maintained in a humidified incubator at 37°C with 5% CO_2_ and cultured in high glucose DMEM medium, supplemented with 10% fetal bovine serum, 100 mg/mL streptomycin and 100 U/mL penicillin. For hypoxic condition, cells were cultured in a tri-gas chamber (GeneScience E500, USA) with 1% O_2_, 94% N_2_ and 5% CO_2_ for 24 h.

### RNA isolation, RNA sequencing and RT-qPCR

Total RNA was extracted using TRIZOL reagent (Solarbio, China) and quantified by Nanodrop2000 spectrophotometer (Thermo Fisher Scientific, USA). Sequencing libraries were generated using NEBNext® Multiplex Small RNA Library Prep Set for Illumina® (NEB, USA) following the manufacturer's recommendations. Library preparation and transcriptome sequencing on an Illumina HiSeq X Ten platform were carried out at Novogene Bioinformatics Technology Co., Ltd. (China). For RT-qPCR, the total RNA was synthesized into cDNA with PrimeScript RT Reagent Kit (Takara, China) in accordance with the manufacturer's protocols. The cDNA was amplified with 2x M5 Ultra SYBR Mixture (Mei5bio, China) on a Bio-Rad CFX96 system (Bio-Rad, USA). The expression of genes was analyzed by 2^-ΔΔCT^, and normalized against β-actin. The primers were purchased from GeneCreate (China), and the sequences are detailed in Supplementary Table S1.

### Transcriptome analysis

The upstream sequencing analysis followed the RNA-Seq standard workflow. After quality control of the sequencing data according to Fastp application, the analysis of differentially expressed genes (DEGs) was done by comparing treatment group and control group samples in each sequencing batch using the R package DESeq2^18^. The thresholds of DEGs identification were |log_2_FC|>1 and P-value<0.05. These DEGs were enrolled in GSEA (Gene set enrichment analysis) analysis through the ClusterProfiler package. Then, the plotting built-in function was used for dotplots and heatmaps presentation.

### Plasmids, siRNA and cell transfection

The full length of *F3* was amplified from U87 cDNA and cloned into pLVX-puro vector by forward primer: 5'-AGATCTCGAGCTCAAGCTTC GAATTC ATGGAGACCCCTGCCTG-3' and reverse primer: 5'-TCCCCTACCCGGTAGAATTATCTAGATTATGAAACATTCAGTGGGGA-3'. The expression plasmids, packaging plasmid containing gag-pol and envelope plasmids containing VSVG were co-transfected into HEK293T cells by using Lipofectamine 3000 (Invitrogen, USA) following the manufacturer's instructions. Lentivirus were collected after 72 h and used for transfection in U87 or LN229 cells. 5 μg/mL puromycin was used to select the stable cell lines according to the antibiotic resistance genes carried by the plasmids. Small inference RNA (siRNA) targeting F3 were synthesized by GeneCreate (China), and the sequences are detailed in Supplementary Table S2. Cell transfection was conducted with Lipofectamine 3000 (Invitrogen, USA) following the manufacturer's instructions.

### Western blotting

GBM cells were collected and lysed in RIPA lysis buffer (Beyotime, China). The protein concentrations were determined using a BCA Protein Assay Kit (Beyotime, China). Proteins were separated by 8-12% SDS-PAGE and then transferred to PVDF membranes (Millipore, Schwalbach, Germany). The membrane was blocked with PBST buffer containing 5% skim milk and incubated with the corresponding primary antibodies at 4 °C overnight. Then, the membrane was washed and hybridized with an HRP-conjugated secondary antibody at room temperature for 2 hours. The signals were detected using ChemiDoc XRS+ (Bio-Rad, USA). The primary antibodies used for western blotting were F3 antibody (ab252918, Abcam), anti-HIF1α antibody (ab179483, Abcam), anti-β-actin antibody (66009-1-Ig, Proteintech), anti-E-cadherin antibody (20874-1-AP, Proteintech), anti-N-cadherin antibody (22018-1-AP, Proteintech), anti-Phospho-NF-κB p65 (3033T, CST), anti-NF-κB p65 (8242T, CST), anti-Bcl-2 (ab182858, Abcam), HRP Goat anti-Rabbit antibody (AS014, Abclonal), and HRP Goat anti-Mouse antibody (SA00001-1, Proteintech). Integrated density of each blotting band was measured by ImageJ (version 1.53q, USA).

### EdU assay

EdU assay was performed using BeyoClick™ EdU-594 kit (C0078S, Beyotime, China) according to manufacturer's instructions. In brief, 2*10^4^ cells were seeded on a cover clip with a diameter of 20 mm in 6-well plate, and incubated in normoxic condition for 4 h. Then the plates were transferred into hypoxic chamber and treated for 10 h. EdU was added into plate for 2 h continuously hypoxic treatment. By fixing with 4% polymethanol and staining with click reaction buffer, cells were then stained by DAPI to mark nucleus. Images were acquired using Andor Revolution WD spinning disc confocal microscope (Oxford Instruments, England).

### Transwell assay

Transwell chambers were used in migration assays. For each replicate, 1*10^4^ cells were plated in 200 μL of serum-free medium on upper chambers which were inserted into a 24-well plate, and 500 μL of medium containing 10% FBS was added to the bottom chamber. After incubation for 24 h under hypoxia, upper chamber cells were gently removed, and the invaded cells in the lower filters were fixed with 4% polymethanol for 10 min, followed by staining with crystal violet for 10 min. The experiments were performed in triplicate and photos were taken by Leica DMi8 microscope (Leica, Germany). Cell counting was conducted using ImageJ (version 1.53q, USA).

### Wound-healing assay

For Wound-healing assay, cells were seeded in 6-well plate at a concentration of 4*10^5^/well. After 24 h, 10 μL pipette tip was used to create a wound scratch. Cells were cultured in 1% O_2_ hypoxic condition for 24 h with serum-free medium. The migration of cells at the wound site was measure by taking images of the wound site at 0 h and 24 h post the wound scratch. ImageJ (version 1.53q, USA) was used to analyze the percentage of wound closure. The experiments were performed in triplicate and photos were taken by Leica DMi8 microscope (Leica, Germany).

### Human Tissue Specimens

LGG (n=5) and GBM (n=5) glioma tissues were obtained from Taihe Hospital, Hubei, China from April 2023 to October 2023. All tissues were preserved in liquid nitrogen immediately after the surgery at Taihe Hospital, Hubei, China. Informed consent was obtained from patients for sample collection, and the study was approved by Ethics Committee of Shiyan Taihe Hospital (Reference number: 2023KS11). Patient information was listed in Supplementary Table S3.

### Analysis of database

The RNA-seq data of different grades and histology of glioma and corresponding clinical information were downloaded from Chinese Glioma Genome Atlas (CGGA) https://www.cgga.org.cn/), The Cancer Genome Atlas (TCGA) (https:// portal. gdc.cancer. gov/) and Rembrandt (GEO accession GSE108474).

GEPIA2 (http://gepia2.cancer-pku.cn/#index) was used to analyze the correlation between the expression of genes in clinical samples 19. The gene expression was quantified by the TPM (transcripts per kilobase per million) of each sample in TCGA GBM tumor datasets. Data were calculated by Spearman's rank correlation coefficient.

### Statistical analysis

Data are shown as the mean ± SEM (error bars) of at least three independent experiments. Statistical analyses were performed with GraphPad Prism 9.0 (GraphPad, USA). Student's t-test or analysis of variance (ANOVA) were applied in the comparison of differences between groups.

## Results

### *F3* is a hypoxia-responsive gene in GBM cells and correlated with glioma grade, histology and patient survival

**Figure 1:**
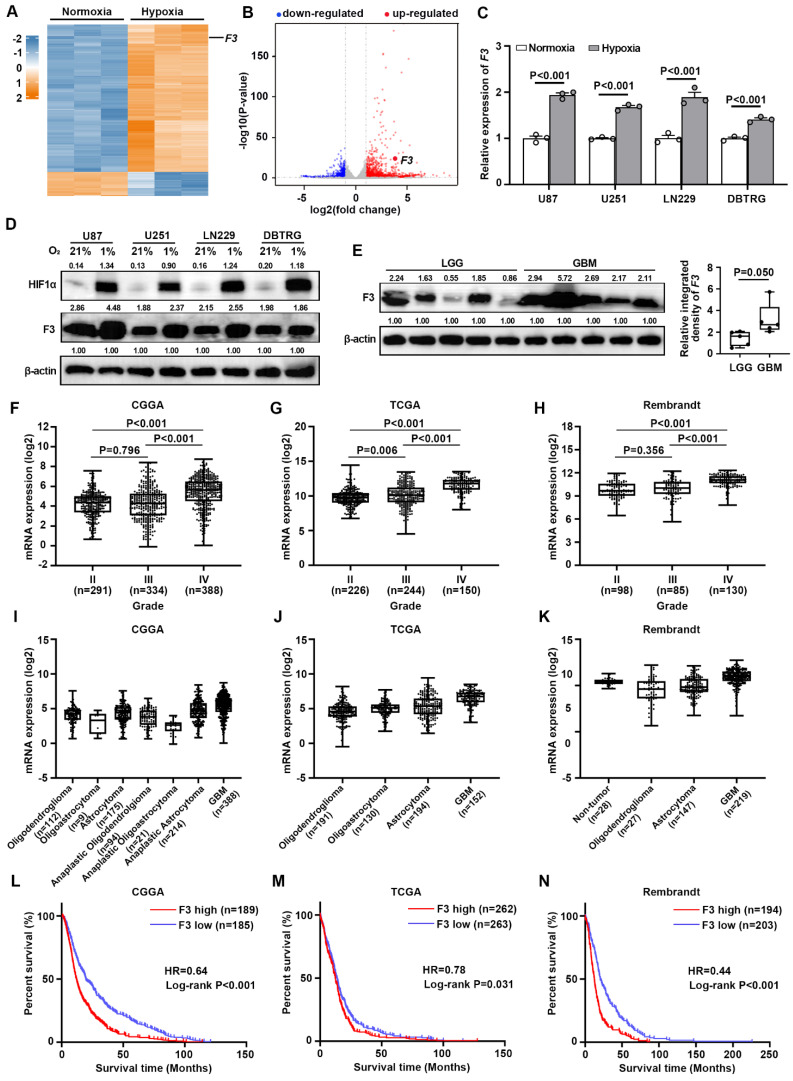
***F3* is a hypoxia-responsive gene in GBM and is related with glioma grade histology and patient survival. (A)** Heatmap shows differentially expressed genes (DEGs) between normoxia and hypoxia in U87 cells on a scale from blue (downregulated) to red (upregulated). **(B)** Volcano plot shows DEGs between normoxia and hypoxia in U87 cells. Horizontal dashed line labels P = 0.05. Vertical dashed lines label |log2fold-change| = 1. **(C)** Validation of the expression level of *F3* in normoxic and hypoxic GBM cells by RT-qPCR. **(D)** Protein levels of HIF1α and F3 in U87, U251, LN229 and DBTRG cells treated under normoxia and hypoxia detected by western blot. Relative integrated density normalized to β-actin was marked above each band. **(E)** Protein levels of F3 in LGG (n=5) and GBM (n=5) clinical samples detected by western blot. Relative integrated density normalized to β-actin is marked above each band. **(F-H)** The expression of *F3* in different grades of glioma from **(F)** CGGA, **(G)** TCGA and **(H)** Rembrandt. **(I-K)** The expression of *F3* in different histology of glioma from **(I)** CGGA, **(J)** TCGA and **(K)** Rembrandt. **(L-N)** Kaplan-Meier survival analysis shows the survival curves of glioma patients with high or low *F3* expression level from **(L)** CGGA, **(M)** TCGA and **(N)** Rembrandt. Data are shown as mean ± SEM (error bars) and were analyzed using Student's t test **(C, E)**, ANOVA **(F-H)** and Log-rank test **(L-N)**.

To investigate potential hypoxia-responsive regulators in GBM cells, RNA-seq (GSE245635) was performed in U87 cells cultured under normoxia (21% O_2_, 74% N_2_ and 5% CO_2_) and hypoxia (1% O_2_, 94% N_2_ and 5% CO_2_) for 24 h. Data analysis showed that 1675 genes were significantly up-regulated and 740 genes were down-regulated under hypoxia with a cut-off criterion of |log2fold-change| > 1.0 and P-value < 0.05 (Figure 1A and B). Among these DEGs, F3 was reported to be a critical regulator of radiation therapy-induced glioblastoma remodeling recently 17. However, the function of *F3* in responses to hypoxia was still unknown. Then, the expression of F3 under hypoxia was further validated in GBM cell lines by RT-qPCR and western blot. Increased expressions of *F3* induced by hypoxia were confirmed in various hypoxia-treated GBM cell lines including U87, U251, LN229 and DBTRG (Figure 1C and D).

To evaluate the clinical significance of F3 in glioma, the protein level of this gene in LGG (n=5) and GBM (n=5) clinical samples by western blot. A remarkable increase of F3 protein was found in GBM samples compared to LGG, indicating that F3 may probably be related to glioma malignancy (Figure 1E). The expression profiles were then analyzed in transcriptional data from CGGA, TCGA and Rembrandt database. Results showed that the expression of *F3* was positively correlated with glioma grade (Figure 1F-H) and higher in GBM compared to other histology of glioma (Figure 1I-K). Kaplan-Meier estimator survival analysis was further applied to compare the prognosis of glioma patients grouped by the mean of *F3* expression level, which indicating that higher expression level of *F3* was companied with worse overall survival (Figure 1L-N). All these results suggested that *F3* is a hypoxia-responsive gene and closely related with the histology and prognosis of glioma.

### *F3* promotes proliferation and migration of GBM cells under hypoxia

**Figure 2:**
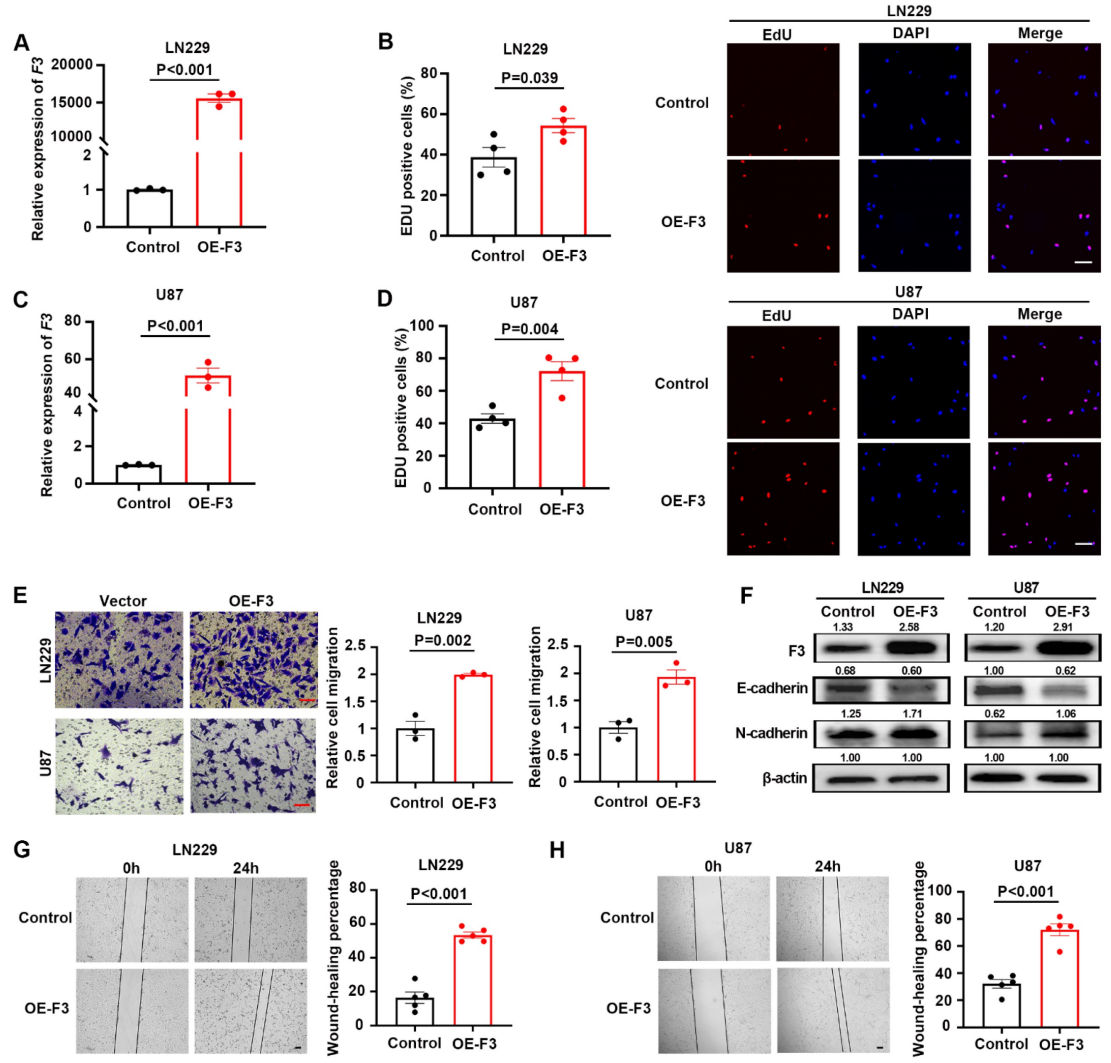
** Overexpression of *F3* promotes proliferation and migration of GBM cells under hypoxia. (A)** Relative expression of* F3* in LN229 cells validated by RT-qPCR. **(B)** The results of EdU assays of LN229 after overexpression of F3 under hypoxia. Quantitative data are shown on left and representative pictures on right. Scale bar: 100 μm. **(C)** Relative expression of *F3* in U87 cells validated by RT-qPCR. **(D)** The results of EdU assays of U87 after overexpression of F3 under hypoxia. Quantitative data are shown on left and representative pictures on right. Scale bar: 100 μm. **(E)** Transwell assays of LN229 and U87 hypoxic cells after overexpression of F3. Representative pictures are on left and quantitative data are on right. Scale bar: 100 μm. **(F)** Representative images of Western Blot show protein levels of F3, E-cadherin and N-cadherin in LN229 and U87 hypoxic cells after overexpression of F3. Relative integrated density normalized to β-actin is marked above each band. **(G and H)** Wound-healing assays of **(G)** LN229 and **(H)** U87 hypoxic cells after overexpression of F3. Representative pictures are on left and quantitative data are on right. Scale bar: 100 μm. Data are shown as mean ± SEM (error bars) and were analyzed using Student's t test **(A-E, G-H)**.

To explore the pathological functions of *F3* in GBM cells, overexpression vector based on lentiviral system was constructed and transfected into LN229 (Figure 2A) and U87 (Figure 2C) cells. The effects of *F3* on the proliferation and migration in were examined under hypoxic conditions. The number of EdU positive cells was significantly increased by overexpression of F3 in LN229 (Figure 2B) and U87 (Figure 2D) compared to controls. In addition, the results of transwell assays showed a remarkable promotion of GBM cell migration by *F3* overexpression (Figure 2E), which was consistent with the wound-healing assays (Figure 2G and H). It is reported that epithelial-to-mesenchymal transition (EMT) is a key biological process in regulating cancer metastasis, thus we investigated whether *F3* could mediate the EMT process. Western blot revealed that overexpression of *F3* led to a decreased expression of the epithelial marker E-cadherin and increased expression of the mesenchymal marker N-cadherin both in LN229 and U87 (Figure 2F).

**Figure 3:**
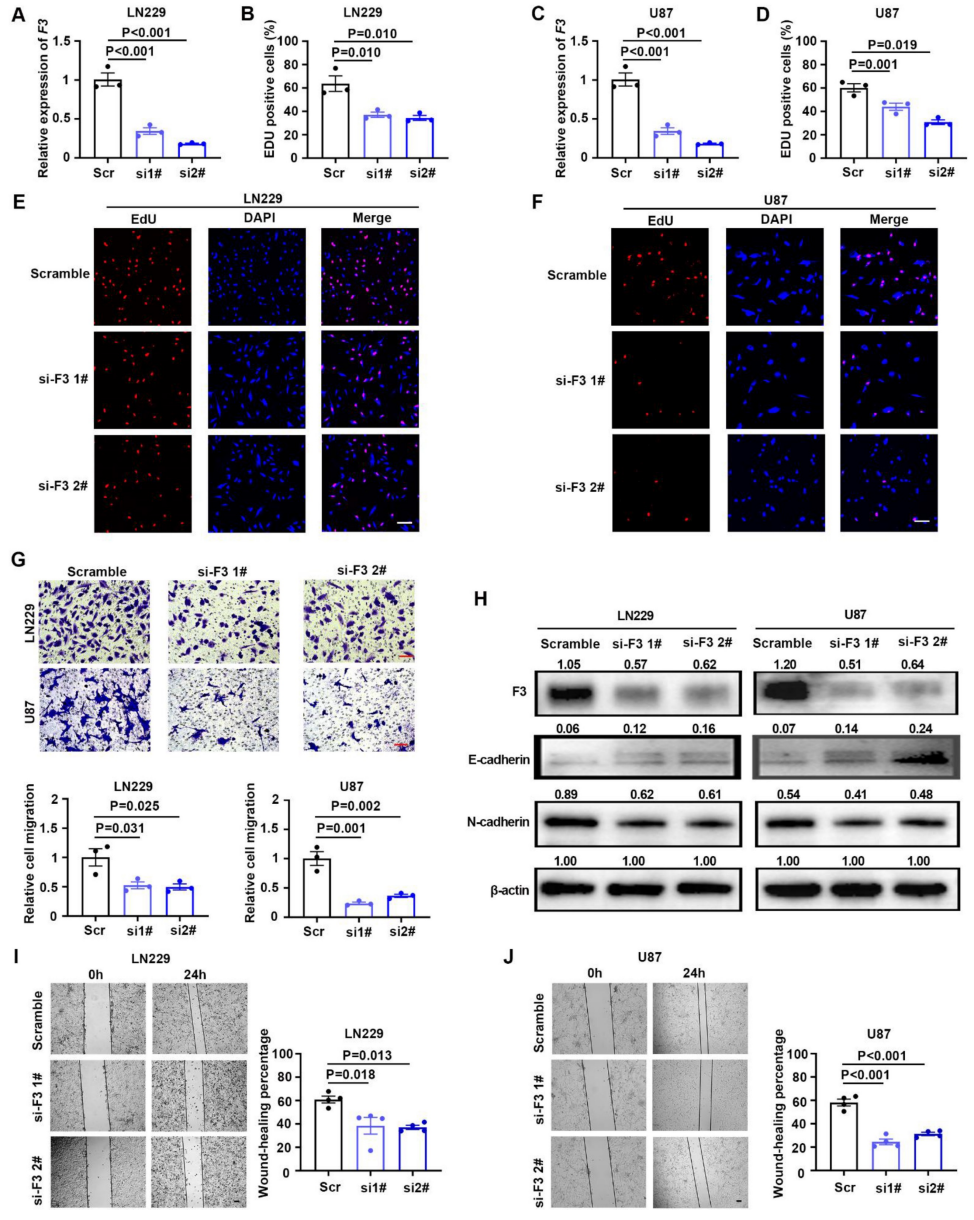
** Knockdown of *F3* inhibits proliferation and migration of GBM cells under hypoxia. (A)** Relative expression of *F3* in LN229 cells transfected with indicated siRNAs targeting F3 compared with the scrambles validated by RT-qPCR. **(B)** Quantitative results of EdU assays after knockdown of F3 in LN229 cells under hypoxia. **(C)** Relative expression of *F3* in U87 cells transfected with indicated siRNAs validated by RT-qPCR. **(D)** Quantitative results of EdU assays after knockdown of F3 in U87 cells under hypoxia. **(E and F)** Representative pictures of EdU assays in **(E)** LN229 and **(F)** U87 hypoxic cells after knockdown of F3. Scale bar: 100 μm. **(G)** Representative pictures (top) and quantitative data (bottom) of Transwell assays on LN229 and U87 hypoxic cells after knockdown of F3. Scale bar: 100 μm. **(H)** Representative images of Western Blot show protein levels of F3, E-cadherin and N-cadherin in LN229 and U87 hypoxic cells after knockdown of F3. Relative integrated density normalized to β-actin is marked above each band. **(I and J)** Wound-healing assays of **(I)** LN229 and **(J)** U87 hypoxic cells after knockdown of F3. Representative pictures are on left and quantitative data are on right. Scale bar: 100 μm. Data are shown as mean ± SEM (error bars) and were analyzed using ANOVA **(A-D, G, I and J)**.

Two siRNAs targeting coding region of F3 were designed and synthesized to knockdown this gene. After transfection of siRNAs, the expression level of F3 were verified by RT-qPCR and western blot in LN229 and U87 cells (Figure 3A, C and H). Knockdown of F3 lead to a significant decrease on GBM cell proliferation under hypoxia (Figure 3B, D, E and F). Moreover, transwell and wound-healing assays further demonstrated that the migration ability of hypoxic LN229 and U87 was suppressed by downregulation of F3 (Figure 3G, I and J). The alteration of E-cadherin and N-cadherin were consistent with the phenotype of cell migration results that E-cadherin was inhibited and N-cadherin was upregulated in after knockdown of F3 in GBM cells. All these results indicated that *F3* is a potential oncogene which could promote cell proliferation and migration of GBM cells.

### *F3* could activate NF-κB signaling pathway in hypoxic GBM cells

**Figure 4:**
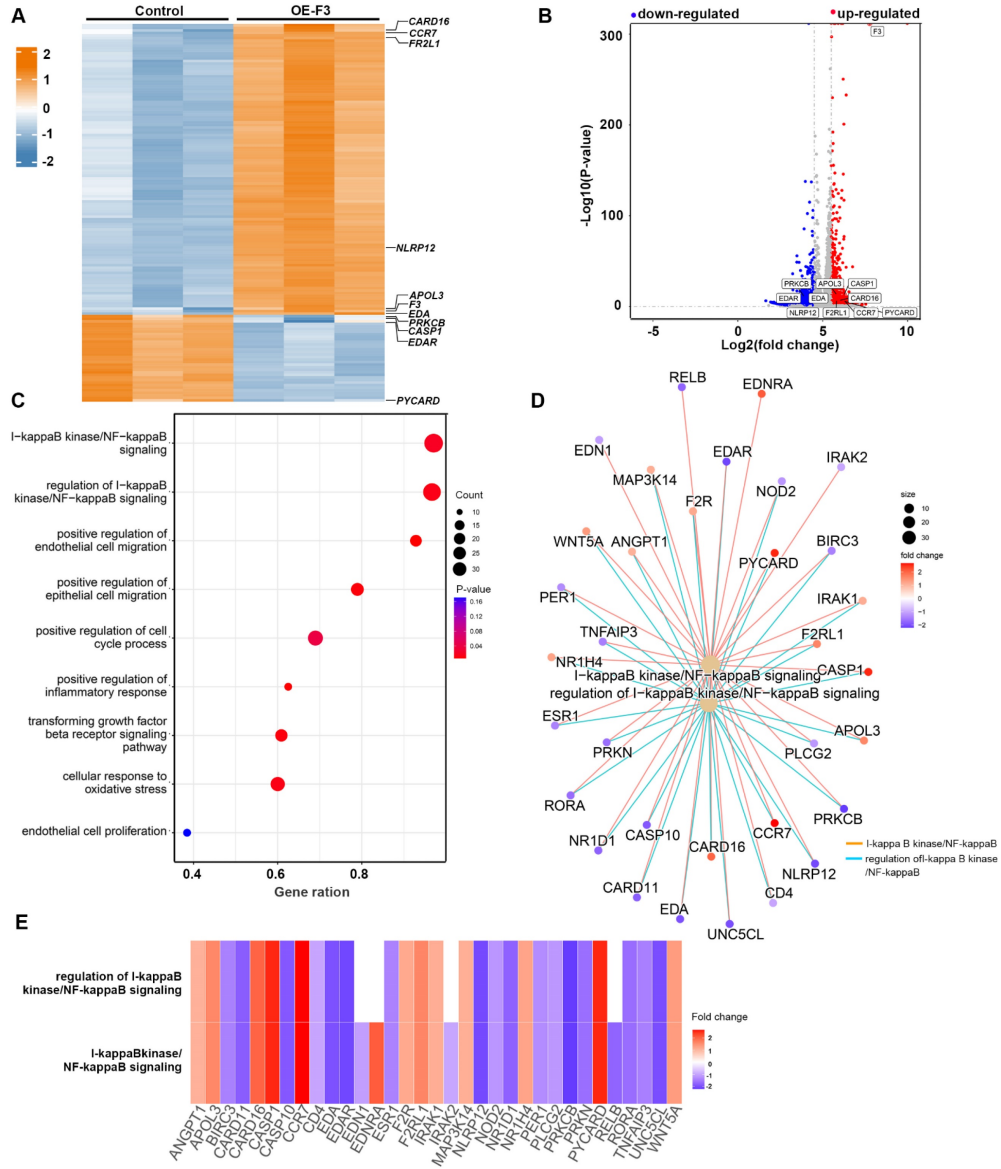
** RNA sequencing profiles for *F3* overexpressed LN229 cells under hypoxia. (A)** Heatmap shows DEGs between OE-F3-LN229 and control cells on a scale from blue (downregulated) to red (upregulated). **(B)** Volcano plot of these DEGs. Horizontal dashed line labels P = 0.05. Vertical dashed lines label |log2fold-change| = 1. **(C)** Gene Set Enrichment Analysis (GSEA) of DEGs in OE-F3-LN229 cells. **(D)** Construction of gene set enrichment regulatory network with NF-κB pathways. **(E)** Heatmap of DEGs enriched in NF-κB pathways.

To uncover the underlying signaling pathways involved in the effects of *F3* on proliferation and migration of GBM cells, RNA-seq was performed in hypoxic *F3* overexpressed LN229 stable cell lines. In total, 483 up-regulated and 2042 down-regulated genes with a cut-off criterion of |log2fold-change| > 1.0 and P-value < 0.05 were identified in OE-F3-LN229 compared to control cells (Figure 4A and B). GSEA revealed that the DEGs were mainly associated with processes related to cell growth and migration (Figure 4C). Among them, one of the most enriched pathways was NF-κB signaling, which is a well-known oncogenic regulator in GBM progression 20. Then, NF-κB associated pathways and differential expressed genes were further analyzed (Figure 4D and E). In the intersection of these two gene subsets, 12 genes were up-regulated and 17 genes were down-regulated, suggesting a dysregulated NF-κB signaling in *F3* overexpressed cells.

**Figure 5:**
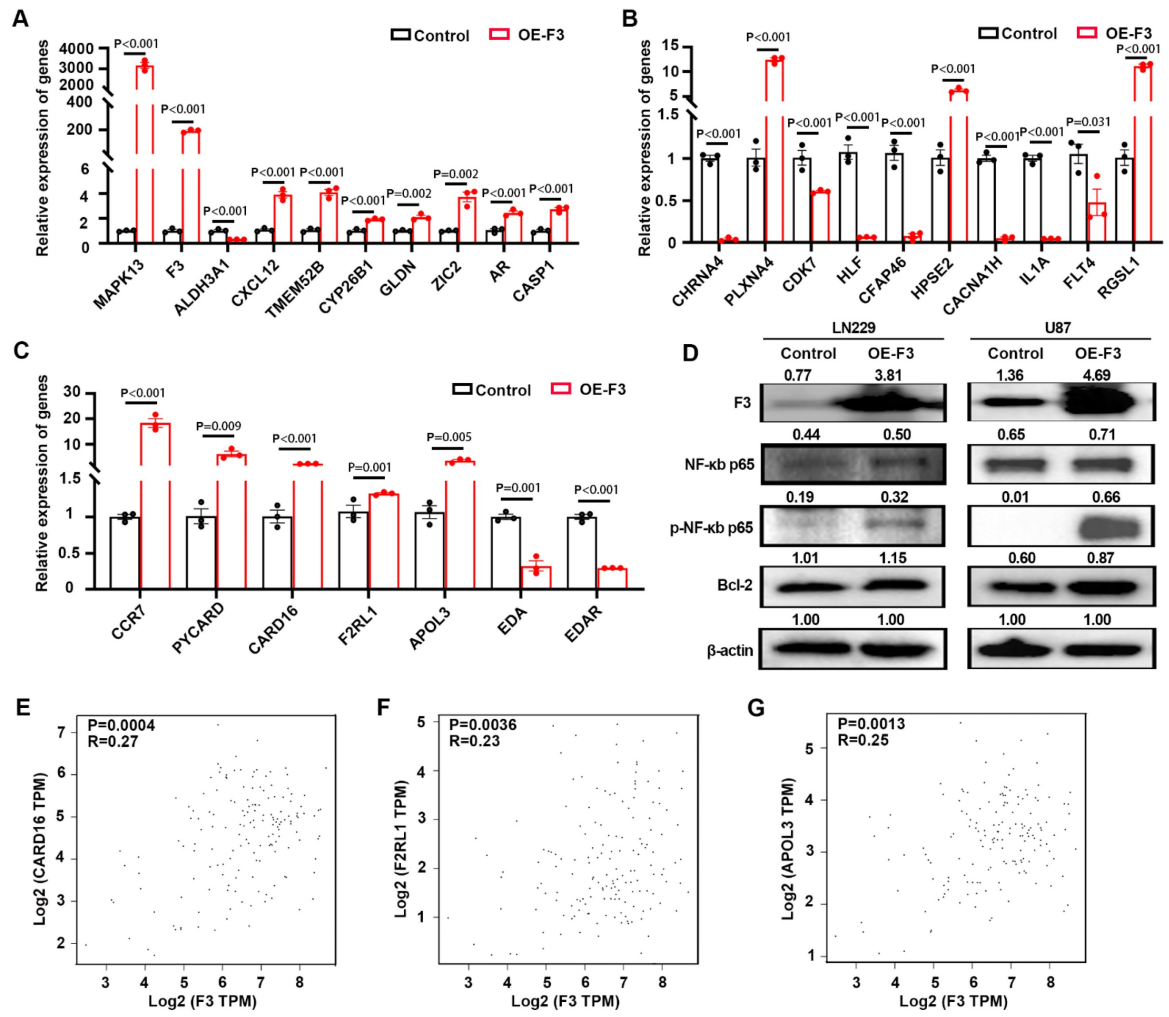
***F3* activates NF-κB signaling pathway in hypoxic GBM cells. (A)** Relative expression level of up-regulated genes in OE-F3-LN229 RNA-seq data validated by RT-qPCR. **(B)** Relative expression level of down-regulated genes in this RNA-seq data validated by RT-qPCR. **(C)** Relative expression level of genes enriched in NF-κB signaling pathway in this RNA-seq data validated by RT-qPCR. **(D)** Representative images of Western Blot show protein levels of F3, NF-κB p65, phospho-NF-κB p65 and Bcl-2 in hypoxic OE-F3-LN229 (left) and OE-F3-U87 (right) cells. **(E-G)** Correlation analysis of F3 and NF-κB downstream genes **(E)** CARD16, **(F)** F2RL1 and **(G)** APOL3 in GEPIA online database based on TCGA GBM dataset. Data were analyzed by Spearman's rank correlation coefficient. Data are shown as mean ± SEM (error bars) and were analyzed using ANOVA test **(A-C)**.

Then, we examined the expression of top DEGs from the RNA-seq profile by RT-qPCR. Nine out of ten up-regulated genes (Figure 5A) and seven down-regulated genes (Figure 5B) had the similar variation tendency with RNA-seq data. The expression levels of DEGs (5 up-regulated and 2 down-regulated genes) related with NF-κB-pathways were also confirmed here, and the results were consistent with RNA-seq data (Figure 5C). Western blot was also performed and the results indicated that overexpression of *F3* increased the phosphorylation level of NF-κB p65 at Ser536, thus regulating the expression of its downstream gene Bcl-2 in LN229 and U87 cells 21 (Figure 5D).

The expression of F3 and NF-κB downstream genes were analyzed by the online tools in GEPIA2 (http://gepia2.cancer-pku.cn/#index) 19. Positive correlations were found between F3 and CARD16, F2RL1, APOL3, which are regulated by NF-κB signaling pathways (Figure 5E-G). Taken together, NF-κB pathway was activated by overexpressing *F3* in hypoxic GBM cells.

### *F3* enhances GBM cell progression by activating NF-κB pathway under hypoxia

**Figure 6:**
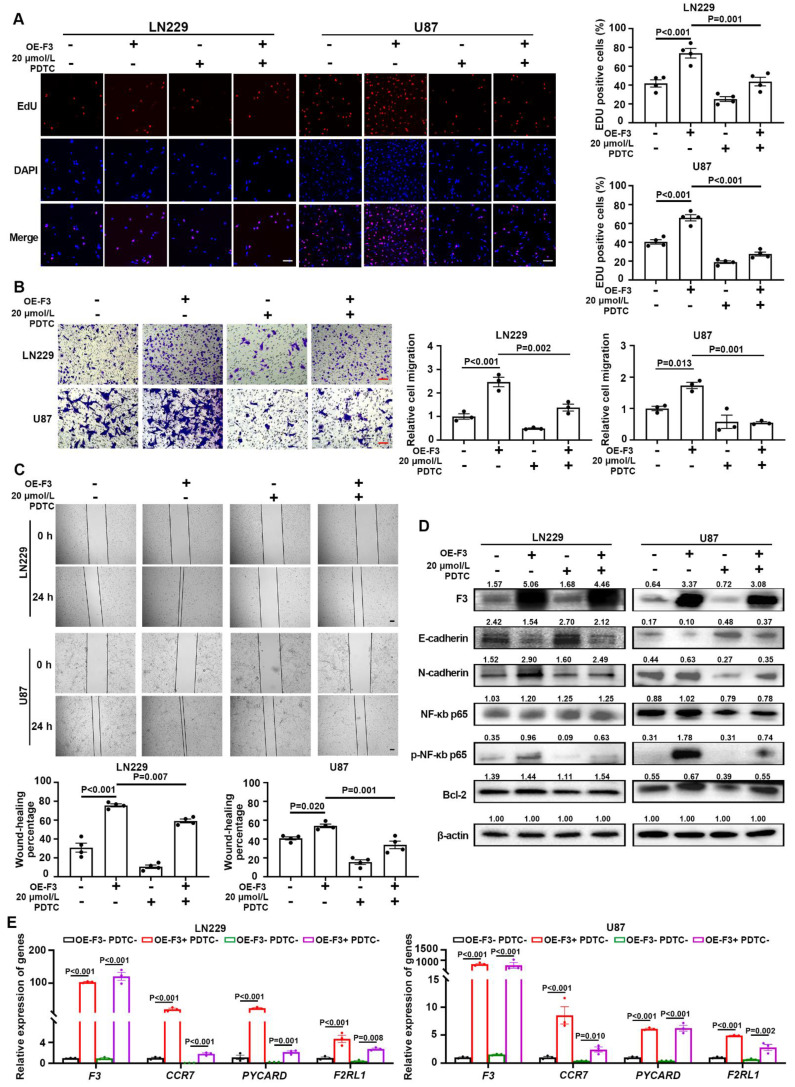
***F3* promotes proliferation and migration of GBM cells through activating NF-κB pathway under hypoxia. (A)** The results of EdU assays in hypoxic OE-F3-LN229 and OE-F3-U87 cells treated with PDTC. Representative pictures are on left and quantitative data are on right. Scale bar: 100 μm. **(B)** Transwell assays of OE-F3-LN229 and OE-F3-U87 cells treated with PDTC. Representative pictures are on left and quantitative data are shown on right. Scale bar: 100 μm. **(C)** Wound-healing assays show hypoxic OE-F3-LN229 and OE-F3-U87 hypoxic cells treated with PDTC. Representative pictures are on top and quantitative data are shown on bottom. Scale bar: 100 μm. **(D)** Representative images of Western Blot show the protein levels of F3, NF-κB p65, phospho-NF-κB p65, Bcl-2 and EMT-related markers **(E-cadherin and N-cadherin)** in hypoxic OE-F3-LN229 cells treated with PDTC. **(E)** Relative expression of NF-κB downstream genes in OE-F3-LN229 and OE-F3-U87 cells after PDTC treatment validated by RT-qPCR. Data are shown as mean ± SEM (error bars) and were analyzed using ANOVA test **(A-E)**.

In order to verify whether the effect of *F3* on GBM progression is dependent on the regulation of NF-κB pathway, hypoxic OE-F3-LN229 and OE-F3-U87 cell lines were treated with pyrrolidinedithiocarbamate ammonium (PDTC), a NF-κB pathway inhibitor 22. As shown in Figure 5A, a significant reduction of cell proliferation was observed in PDTC-treated cells under hypoxia. Moreover, PDTC treatment rescued the increased migrated cell numbers induced by *F3* overexpression in hypoxic LN229 and U87 cells (Figure 6B). Similar results were also observed in the wound-healing assays (Figure 6C). E-cadherin was up-regulated, while N-cadherin was down-regulated after PDTC incubation, indicating an inhibition of EMT process (Figure 6D). These results suggested that blocking NF-κB pathway partly rescued the enhanced proliferation and migration of GBM cells induced by *F3*.

Subsequently, the phosphorylation level of p65 was analyzed by western blot. After PDTC treatment, the protein level of phosphorylated p65 along with its downstream gene Bcl-2, were decreased in OE-F3-LN229 and OE-F3-U87 cells, while total p65 had no noticeable change (Figure 6D). The mRNA expression levels of NF-κB downstream genes further confirmed our hypothesis (Figure 6E). All these findings indicated that *F3* activates NF-κB pathway through upregulating the phosphorylation of p65 at Ser536, thus promoting GBM cell progression.

## Discussion

Hypoxia is one of the hallmarks in tumor microenvironment due to excess malignant proliferation and limited angiogenesis. Tumor cells need to adapt to hypoxic microenvironment through transcriptional reprogramming of hypoxia-responsive genes to ensure energy production and cell survival 23. The brain has lower concentration of oxygen than other organs which is at a concentration of 3 - 4.5%, therefore GBM is more susceptible to hypoxia than other tumors 4, 24. In this study, *F3* was identified as a hypoxia-responsive gene that was significantly up-regulated in hypoxic U87 cells by RNA-seq. The induction of this gene under hypoxia could be observed in a series of GBM cell lines. Then, we analyzed the expression level of *F3* in clinical samples from CGGA, TCGA and Rembrandt databases, and found that highly expressed *F3* is correlated with poor survival of patients, which implied an oncogenic role of *F3* in tumor progression of GBM. The results of EdU, Transwell and wound-healing assays reaved that *F3* could promote the proliferation and migration of hypoxic U87 and LN229 cells *in vitro*. These results indicated that *F3* is a potential oncogene promoting GBM progression under hypoxia.

As the core regulator of hypoxia, hypoxia-induced 1 α subunit (HIF1α) activates a series of gene transcription and leads to a more aggressive and metastatic phenotype of GBM 25-30. For example, the expression of PRMT2 is activated by HIF1α causing a cell migration and accelerating GBM progression 31. In this work, we identified *F3* was a hypoxia-induced gene that was significantly up-regulated under hypoxia in GBM cells. However, the mechanism underlying the dysregulation of *F3* in this process is still unknown. According to the analysis based on JASPAR database, 20 putative HIF1α binding sites were predicted in the promoter region of *F3* (GRCh38.p14, chromosome 1 - NC_000001.11: c94543759-94541660) with relative profile score threshold 80%, indicating that HIF1α probably binds to the promoter of *F3* and directly induces the expression of this gene. Nevertheless, whether the dysregulation of *F3* under hypoxia was dependent on HIF1α signaling still needs to be further investigated.

NF-κB is a small menagerie of closely related protein dimers that inhibits E-cadherin and causes cell migration in GBM 32. The phosphorylation of at p65 Ser536 are considered as a marker of activation of NF-κB, which promote proliferation and metastasis of GBM cells 20, 33, 34. For example, Wang *et al.* found that the stimulation of IL-17 greatly increased the phosphorylation of NF-κB/p65 at Ser536, enhanced the GBM cell progression 35. He *et al.* treated U251 cells with cortistatin, which weakens the activation of NF-κB/p65, thus suppressing TMZ resistance by regulating MGMT 36. Jeon *et al.* found that *F3* could regulate GBM radio resistance and recurrence and phosphorylated p65 was elevated in *F3*-positive GBM cells after radiotherapy 17. Consistent with these previous studies, NF-κb/p65 signaling could also be activated by *F3* overexpression in GBM cell under hypoxia in our study. Besides, we found that administration of PDTC effectively rescues the enhanced proliferation and migration induced by over-expressed *F3* in LN229 cells. Collectively, we concluded that *F3* promotes GBM cell progression through activating NF-κB/p65 axis. However, how *F3* regulates the phosphorylation of p65 under hypoxia is still a mystery, and remains to be further explored.

The DEGs in OE-F3-LN229 cells were enriched in pathways associated with cell growth and metastasis, including transforming growth factor beta receptor (TGFBR) signaling (Figure 3). The binding of TGF-β to its receptors can activate noncanonical TGF-β signaling by stimulating a series of kinases such as MAPKs 37. Interestingly, we observed that overexpression of *F3* also increases the expression of MAPK13 (Figure 4A), which indicating that *F3* may also trigger the TGFBR signaling. As previous reported, TGFBR2 could physically interact with IL1R, resulting in the activation of NF-κB-p65 38, 39. All these results indicated that *F3* may mediate the crosstalk between TGF-β and NF-κB signaling.

As a summary, this study demonstrated the role of *F3* in proliferation and migration of GBM cells responding to hypoxia. Overexpression of *F3* induced the alteration of a series DEGs, which were enriched in NF-κB-related pathways. PDTC treatment could partially rescue the phenotype induced by *F3* overexpression in LN229, which indicated that the effect of *F3* on GBM progression is dependent on the regulation of NF-κB pathway. All these data implicated that *F3* promotes the proliferation and migration of GBM cells via the activation of NF-κB signaling under hypoxia and may provide new insight into the treatment and diagnosis of GBM.

## Supplementary Material

Supplementary tables.

## Abbreviations

GBM: glioblastoma multiforme
TMZ: temozolomide
NF-κB: nuclear factor kappa B
HIF1α: hypoxia-induced factor 1 α
Bcl-2: B-cell lymphoma-2
TCGA: The Cancer Genome Atlas
CGGA: Chinese Glioma Genome Atlas
OE: overexpression
EMT: epithelial-to-mesenchymal transition
GSEA: Gene Set Enrichment Analysis
DEGs: differentially expressed genes
PDTC: pyrrolidinedithiocarbamate ammonium
