# Supplementary material for: Hypoxia-responsive gene F3 Promotes GBM Cell Proliferation and Migration through Activating NF-κB/p65 Signaling Pathway
Source: J Cancer. 2024 Jun 17;15(14):4477–89. doi: 10.7150/jca.97357 (PMC11242329; doi:10.7150/jca.97357)
Supplement: Supplementary file 1 — Supplementary tables. [file jcav15p4477s1.pdf]

Supplementary Table S1. Primers for RT-qPCR.

Name	Sequence	
qF3-F	5'-3'	GGCGCTTCAGGCACTACAA
qF3-R	5'-3'	TTGATTGACGGGTTTGGGTTC
β-actin-F	5'-3'	TCCCTGGAGAAGAGCTACG
β-actin-R	5'-3'	GTAGTTTCGTGGATGCCACA
qMAPK13-F	5'-3'	TGAGCCGACCCTTTCAGTC
qMAPK13-R	5'-3'	AGCCCAATGACGTTCTCATGC
qALDH3A1-F	5'-3'	TGGAACGCCTACTATGAGGAG
qALDH3A1-R	5'-3'	GGGCTTGAGGACCACTGAG
qCXCL12-F	5'-3'	ATTCTCAACACTCCAAACTGTGC
qCXCL12-R	5'-3'	ACTTTAGCTTCGGGTCAATGC
qTMEM52B-F	5'-3'	GAAGTGACCGTCATTGCTTTCG
qTMEM52B-R	5'-3'	CTGTGAAGCGACTCATGTGAA
qCYP26B1-F	5'-3'	GGCAACGTGTTCAAGACGC
qCYP26B1-R	5'-3'	TGCTCGCCCATGAGGATCT
qGLDN-F	5'-3'	TGGTGCCGATCCGAGTGAT
qGLDN-R	5'-3'	GGCTGTCCATCCAATCCGTT
qZIC2-F	5'-3'	GCGCAACTCCACAACCAGTA
qZIC2-R	5'-3'	TGCCGCATATAGCGGAAAAAG
qAR-F	5'-3'	CCAGGGACCATGTTTTGCC
qAR-R	5'-3'	CGAAGACGACAAGATGGACAA
qCASP1-F	5'-3'	TTTCCGCAAGGTTCGATTTTCA
qCASP1-R	5'-3'	GGCATCTGCGCTCTACCATC
qCHRNA4-F	5'-3'	GGAGGGCGTCCAGTACATTG
qCHRNA4-R	5'-3'	GAAGATGCGGTCGATGACCA
qPLXNA4-F	5'-3'	GTCATTTGTCACATTCCGAGGA
qPLXNA4-R	5'-3'	GCTTGTAAATCCGATTGACGGC
qCDK7-F	5'-3'	ATGGCTCTGGACGTGAAGTCT
qCDK7-R	5'-3'	GCGACAATTTGGTTGGTGTTC
qHLF-F	5'-3'	CCACCTTTATCCCGCCTCC
qHLF-R	5'-3'	TTTACTAAATGCGTCTTCGTGGT
qCFAP46-F	5'-3'	CAAAGGAGAACCGAGGTACTACT
qCFAP46-R	5'-3'	ACTCAGCACGTTTATGATTTGGG
qHPSE2-F	5'-3'	ATGGCCGGGCAGTAAATGG
qHPSE2-R	5'-3'	GCTGGCTCTGGAATAAATCCG
qCACNA1H-F	5'-3'	ATGCTGGTAATCATGCTCAACTG
qCACNA1H-R	5'-3'	AAAAGGCGAAAATGAAGGCGT
qIL1A-F	5'-3'	TGGTAGTAGCAACCAACGGGA
qIL1A-R	5'-3'	ACTTTGATTGAGGGCGTCATTC
qFLT4-F	5'-3'	TGCACGAGGTACATGCCAAC

Name	Sequence	
qFLT4-R	5'-3'	GCTGCTCAAAGTCTCTCACGAA
qRGSL1-F	5'-3'	CAACCACTAGGAGGGAGATCC
qRGSL1-R	5'-3'	TCATCTTGGTGTGGGTGTAAAAG
qCCR7-F	5'-3'	TGAGGTCACGGACGATTACAT
qCCR7-R	5'-3'	GTAGGCCCACGAAACAAATGAT
qPYCARD-F	5'-3'	TGGATGCTCTGTACGGGAAG
qPYCARD-R	5'-3'	CCAGGCTGGTGTGAAACTGAA
qCARD16-F	5'-3'	TGGGTGAAGGTACAATAAATGGC
qCARD16-R	5'-3'	AGCTCGGGTCTTATCCATAACT
qF2RL1-F	5'-3'	CAGTGGCACCATCCAAGGAA
qF2RL1-R	5'-3'	CAGGGCCATGCCGTTACTT
qAPOL3-F	5'-3'	GGGACGAGTCTGGCCCTTA
qAPOL3-R	5'-3'	TCAATCGGTCAATGCTGGTTG
qCARD11-F	5'-3'	GGACGCCTTGTGGGAGAATG
qCARD11-R	5'-3'	TCAATGACCTTACACTGACGC
qEDA-F	5'-3'	AGATGGCCCAGTTAAAAACAAGA
qEDA-R	5'-3'	CAGGTGGTCCCATAACAGTTG
qEDAR-F	5'-3'	CAGCCCGAGCGGAATACTC
qEDAR-R	5'-3'	CCGTAGCCACAGGACAGGTA
qNLRP12-F	5'-3'	GGGGCTTGTCAGGAGATGG
qNLRP12-R	5'-3'	AGTCCCTGGCATAGTAACCTC
qUNC5C-F	5'-3'	TGGGACTGGGATACTTGCTG
qUNC5C-R	5'-3'	ACAGTACAGGTTCACAGGCTTAT

Supplementary Table S2. siRNAs targeting F3.

Name	Sequence	
si-Scramble	5'-3'	UUCUCCGAACGUGUCACGUTT
si-F3 1#	5'-3'	GCUCUGAGCUAGGCAAUUU
si-F3 2#	5'-3'	GCGCUUCAGGCACUACAAA

Supplementary Table S3. Patient information for Western Blot

Tissue number	Gender	Age	Grade
P1	male	61	II
P2	female	56	II
P3	male	44	II
P4	male	34	II
P5	male	47	II
P6	male	70	IV
P7	male	54	IV
P8	female	54	IV
P9	male	29	IV
P10	female	59	IV
